# Incidence and Length of Sickness Absence among Hierarchical Occupational Classes and Non-Wage-Earners: A Register Study of 1.6 Million Finns

**DOI:** 10.3390/ijerph18020501

**Published:** 2021-01-09

**Authors:** Jenni Blomgren, Sauli Jäppinen

**Affiliations:** 1 Research Unit, The Social Insurance Institution of Finland, P.O. Box 450, 00056 KELA, Helsinki, Finland; 2 Analytics Unit, The Social Insurance Institution of Finland, P.O. Box 450, 00056 KELA, Helsinki, Finland; sauli.jappinen@kela.fi

**Keywords:** sickness absence, social determinants, socioeconomic, occupational class, entrepreneurs, unemployed

## Abstract

Socioeconomic differences in sickness absence are well known, but previous studies have tended to focus on wage earners only. This study examined incidence and length of sickness absence comparing the employee groups of upper and lower non-manual employees and manual workers, but also entrepreneurs, the unemployed and other non-wage-earners. The study utilized register data on a nationally representative 70% sample of Finns aged 25–62 at the end of year 2012 (N = 1,615,352). Sickness absence spells compensated by sickness allowance and initiated during 2013 were retrieved from the register of the Social Insurance Institution of Finland (SIIF) and followed until the end of each episode and linked to socio-demographic covariates collected from the registers of the SIIF and of Statistics Finland. Zero-inflated negative binomial regression was used in multivariate models. After adjusting for age, marital status, region and income, there were clear differences in the occurrence and length of sickness absence across socioeconomic groups. Compared to upper non-manual employees, lower non-manual employees and especially manual workers had higher cumulative annual incidence of sickness absence among both men and women, but the entrepreneurs, the unemployed and other non-wage-earners had a clearly higher expected number of sickness absence days. Results varied by diagnostic group. The results highlight the importance of different types of preventive measures for reducing the occurrence of sickness absence and for preventing prolongations of sickness absence spells in different socioeconomic groups.

## 1. Introduction

Sickness absence incurs significant costs for the employers, for the individuals and their families and for the society as a whole in terms of lost productivity, lost income, and payments of sickness benefits [1,2]. In Finland, around two thirds of employees have at least some sickness absence during one year’s time [3], and about 4% of theoretical yearly working time (i.e., regular working time in accordance with an agreed system of working hours and including annual leave) are estimated to be lost due to sickness absence [4]. Each year, around 10% of non-retired Finns have long sickness absence spells that exceed 10 working days, entitling to sickness allowance paid by the Social Insurance Institution of Finland [5]. Sickness absence overall, and long sickness absence in particular, is strongly associated with poor health, disability retirement and even mortality [6,7,8,9,10,11]. Knowledge of the determinants of sickness absence and of the accumulation of sickness absence days is important to be able to target effective measures that aim to prevent both incidence and prolongation of sickness absence and to curb its harmful consequences.

Several previous studies conducted in different countries have generally shown that socioeconomic status is associated with the risk of sickness absence: those in a lower occupational class have more sickness absence than those in a higher occupational class [12,13,14,15,16,17,18,19,20,21,22,23]. Moreover, the observed socioeconomic differences depend on the diagnostic group: for example, of the two largest diagnostic groups of sickness absence, musculoskeletal diseases tend to show large socioeconomic differences while mental disorders show smaller and partly divergent socioeconomic patterns [20,21]. Furthermore, women have more sickness absence than men do, but the socioeconomic gradient—i.e., the increased likelihood of being sickness absent among persons in lower socioeconomic positions—tends to be stronger among men than among women [19]. These results warrant investigating men and women separately and looking at diagnostic differences in addition to all-cause sickness absence.

However, previous studies on the socioeconomic gradient of sickness absence have mainly focused on wage earners only. This is understandable since most data sets used in sickness absence studies are based on employed cohorts. However, also groups not in a wage earner position within the working-age populations are susceptible to poor work ability and, in consequence, to sickness absence. In many benefit systems, such as in the Scandinavian welfare states of Finland and Sweden, also the entrepreneurs may be listed as sickness absent and receive sickness benefits if their poor work ability prevents them from working. The unemployed may receive sickness benefits if their poor work ability prevents them from seeking and accepting employment. Furthermore, also other groups in non-wage-earner positions may be eligible to receive sickness allowances. Thus, in addition to the hierarchical occupational classes that have most often been studied (often using the classification of upper and lower non-manual employees and manual workers), sickness absence research is needed also concerning the non-wage-earners in the working-age populations, and especially concerning the groups of entrepreneurs and unemployed. The entrepreneurs are often seen as the backbone of the society in terms of creating jobs, and thus monitoring their work ability is crucial. The unemployed, in turn, are an important labor reserve, and maintaining and monitoring also their work ability is justified. Previous studies have shown that the self-employed may have a higher risk of transitioning to sickness absence compared to the wage earners overall [24]. It is also known that unemployed individuals have on average poorer psychological and physical health than employed individuals do [25], and they may have an elevated risk of later sickness absence [26]. However, we are not aware of sickness absence analyses that simultaneously include both wage earner and non-wage-earner groups—and more specifically, analyses that include the entrepreneurs and the unemployed as groups of their own. More research is needed assessing the full socioeconomic spectrum and including the total working-age population susceptible to sickness absence.

In addition to focusing on employed populations only, previous studies have often concentrated on limited measures of sickness absence, most often measuring annual proportions of persons with sickness absence spells. In order to deepen previous knowledge, the aim of this study was to examine both the incidence of sickness absence and the length of absence spells using nationally representative register-based data on the Finnish working-age population. Sickness absence was examined both among hierarchical occupational classes (upper and lower non-manual employees and manual workers) and among the entrepreneurs and the unemployed, as well as among the remaining group of other non-retired working-age persons, i.e., the rest of the non-wage-earners. In addition to all-cause sickness absence, sickness absences in the two largest diagnostic groups, i.e., musculoskeletal diseases and mental disorders, were assessed separately.

## 2. Materials and Methods

### 2.1. Study Design and Participants

The study population was retrieved from the population data file of the Social Insurance Institution of Finland and consists of a 70% random sample of the Finnish population between ages 25 and 62 at the end of year 2012 (total N = 1,888,481). The lower age limit was set at 25 years in order to measure socioeconomic status more reliably than would be possible among younger adults. Furthermore, since measurement of sickness absence is meaningful and relevant only concerning those not retired and old age retirement was possible at age 63, the upper age limit was set at 62. Those who were retirees at the end of 2012 (N = 147,926) were removed from the analyses since the retired (disability pensioners and old-age pensioners) are not eligible for sickness allowance, our measure for sickness absence (see below Section 2.2). The data are nationally representative of the non-retired Finnish population in the above-mentioned age span.

The data set was constructed through linkages of the population and sickness allowance registers of the Social Insurance Institution of Finland (permission Kela 59/522/2015) and of the register of occupational class from Statistics Finland (permission TK-53-1106-15). The data were linked at the Social Insurance Institution of Finland, using the personal identity codes assigned to every Finnish citizen, after which the personal identity codes were removed from the data set. The researchers only had access to pseudonymized data. As the study used only secondary data retrieved from registers and no human subjects were contacted to collect the data, no ethics approval was required according to Finnish law and practices [27]. Conventions of good scientific practice, data protection and information security have been applied in analyzing the data and presenting the results.

### 2.2. Measurement of Sickness Absence

Sickness absence was measured through recipiency of sickness allowance. The Social Insurance Institution of Finland can pay sickness allowance to an individual whose disease or injury has caused reduced work capacity for more than 10 working days (i.e., calendar days excluding Sundays and midweek holidays—the so-called waiting period). Thus, since sickness absence was measured through receipt of sickness allowance, all sickness absence episodes that were included in this study were rather long-term, at least 12 calendar days long absences. A physician’s certificate is required to obtain the benefit. If the person is employed, the employers compensate the first days through sick pay [28]. Sickness allowance can be paid up to approximately one year, after which a disability pension may be considered if the work disability continues.

Register data was used on all new sickness allowance episodes that started during 2013, with their beginning and end dates as well as diagnoses. Overlapping and consecutive episodes of sickness absence in the original data were combined. Diagnosis of each sickness absence episode was recorded based on the International Classification of Diseases, 10th revision (ICD-10) [29]. In addition to analyzing all-cause sickness absences, we also examined separately the two most common disease groups of sickness absence: diseases of the musculoskeletal system and connective tissue (ICD-10 codes M00–M99), and mental and behavioral disorders (ICD-10 codes F00–F99).

The length of each separate sickness absence episode was first calculated from the beginning date to the end date of the absence episode. Then, the total number of sickness absence days of an individual was calculated as a sum of length of all episodes and separately for the two diagnostic groups of musculoskeletal and mental diagnoses. Full information on the length was used also of those sickness absence episodes that extended until year 2014. Thus, if an absence episode started in 2013 but ended in 2014, we included also the absence days in year 2014 in the calculation of the length of that episode.

Working-age persons are eligible for sickness allowance only if they have not already accumulated the maximum amount of payable allowance days, totaling to approximately one year, but a one-year consecutive period of non-use of allowance resets the counter. Thus, some absence spells in 2013 may have been cut short because the allowance days had already mostly been used up during the previous year. In order to overcome this problem and to focus on the incidence of truly new sickness absence episodes among previously healthy persons, the analyses were restricted to those who had received no prior sickness allowance during one year’s time before the onset of their first absence episode in 2013. For persons who had no new sickness absence spells in 2013, the one year’s healthy period was assessed from 1 July 2013 backwards. The same requirement of one healthy year was applied irrespective of the diagnosis of the first occurring sickness absence episode. After including only those with no prior sickness allowance in the recent history, 125,203 persons were dropped, and the final sample used in the analyses included 1,615,352 persons (822,766 men, 792,586 women).

### 2.3. Occupational Class and Socio-Demographic Covariates

The variable of main interest in the analyses was occupational class. Information on occupational class at the end of 2012 was retrieved from the register of Statistics Finland. The persons were classified into six groups according to the classification of Statistics Finland [30]: first, three employee groups of upper non-manual employees, lower non-manual employees and manual workers, and second, the non-employee groups of entrepreneurs (including the self-employed and owners of companies with salaried employees), unemployed, and other non-wage-earners. Some examples of occupations belonging to the group of upper non-manual employees are directors and managers, white-collar qualified specialists, teachers and physicians. Lower non-manual employees include, for example, technicians, nurses, childminders and police officers, and manual workers include, for example, construction workers, transport workers, mechanics and cleaners [30]. The unemployed group consisted of persons with at least 6 months of consecutive unemployment; thus, sporadic unemployment did not suffice to be included in this group. The last group of others consisted of students and other persons outside the labor force who could not be classified into the other previously mentioned categories, or whose occupational class was unknown.

In addition, several individual-level socio-demographic covariates were retrieved from the registers of the Social Insurance Institution of Finland. The covariates included gender, age (used in age bands 25–34, 35–44, 45–54 and 55–62 years), marital status (married, never married including those with unknown marital status, and divorced or widowed) and region of residence (four large regions based on geographic location) at the end of 2012, and taxable yearly income during 2012 (including both earned income and capital income, classified into quintiles). These covariates were taken into account since various socio-demographic variables are important predictors of sickness absence and may also confound the association between occupational class and disability [10,11,12,13,14,21].

### 2.4. Statistical Methods

The outcomes of the study were (1) the incidence of at least one new sickness absence episode in the study population during 2013, corresponding to the cumulative incidence measure suggested by Hensing [31] and (2) length of sickness absence, measured as the total number of sickness absence days in absence episodes that started during year 2013 among those who had new episodes in 2013 [31]. Since both incidence and length of sickness absence are different among men and women, and there are clear differences in the association of occupational class with sickness absence by gender [19], all analyses were performed separately for men and women. Descriptive statistics were obtained for all variables on the incidence of sickness absence and the number of absence days.

When linked to population data, the data on the number of sickness absence days in our data have an excess number of zeros and are severely over-dispersed: a large proportion of persons are not sickness absent at all so that they would receive sickness allowance, while among those who are, the distribution is strongly right-skewed. Partly, not being absent may be related to never being ill. Additionally, not being absent may be related to other reasons, such as sickness presenteeism i.e., being at work while sick, non-willingness to get medical treatment and get a doctor’s certificate for sickness absence, or some other reasons. Both the excess number of zeros and the overdispersion need to be taken into account in the analytic strategy. Main options for modeling this type of count data are a simple Poisson model, a simple negative binomial model, a zero-inflated Poisson model, and a zero-inflated negative binomial (ZINB) model [32,33]. According to statistical tests, our data were severely over-dispersed. The Vuong test supported use of ZINB models over standard negative binomial regression models [34]. Furthermore, the likelihood ratio test showed preference of zero-inflated negative binomial models over zero-inflated Poisson models. Thus, we chose zero-inflated negative binomial regression models as the analytic strategy to allow for both excess zeros and for overdispersion [35,36,37,38]. Additionally, several other studies on sickness absence have utilized this modeling strategy [33,39,40].

The ZINB models allow for modeling separately the excess zeros and the number of days among the non-zero group. Thus, the results are divided into two parts. The first part (the logit part) predicts the probability of having/not having sickness absence (i.e., membership in the zero group), expressed as odds ratios (OR) with their 95% confidence intervals (CI). For easier interpretation of the results, the odds ratios were reversed to denote the relative odds of belonging to the non-zero group instead of being in the always zero group. This reparameterization is mathematically equivalent with the original model [33]. The second part of the model (the negative binomial part) predicts the number of sickness absence days among those who had sickness absence (i.e., among the not always zero group), expressed as incidence rate ratios (IRR) with their 95% CIs. The IRRs can be interpreted as how many times more or less sickness absence days those in the occupational class in question are expected to have compared with the reference group [41]. In our analyses, the upper non-manual employees served as the reference group.

Robust standard errors were used to control for omitted individual heterogeneity [38]. Models were adjusted for all covariates. The analyses were carried out with the Stata statistical package, version 13 [42].

## 3. Results

### 3.1. Characteristics of Participants

The distributions of the individual-level variables are shown in Table 1. Women were slightly older than men were, more often married, divorced or widowed and less often never married, lived slightly more often in the Capital and Uusimaa region, and were more often than men in the lower income quintiles. In terms of occupational class, compared to women, men were about twice as often manual workers (M: 31%; W: 14%) and also clearly more often entrepreneurs or unemployed, while women were more often than men in the group of lower non-manual employees (M: 19%; W: 43%).

### 3.2. Descriptive Results on the Incidence and Length of Sickness Absence

The associations of the socio-demographic covariates with all-cause sickness absence are presented in Table 1. 7% of men and 10% of women in the study cohort had at least one sickness absence episode compensated by sickness allowance that started during year 2013 (Table 1). Even though the cumulative incidence of sickness absence was higher among women than men, the mean number of sickness absence days among those who had any sickness absence episodes was higher among men (84 days) than among women (71 days). Higher age was associated with both higher cumulative incidence and longer length of sickness absence episodes among both men and women. Both incidence and length of sickness absence were higher than average in the group of divorced or widowed persons, and among those living in the Northern and Eastern parts of the country (i.e., geographically most remotes areas). Cumulative incidence of sickness absence was highest in the middle-income categories and lowest both in the high end and in the low end of the income distribution. However, the association of the length of sickness absence with income showed a different pattern: the lower the income, the higher the number of sickness absence days. The number of days was particularly large among those with lowest income (Table 1).

Differences in sickness absence in terms of occupational class were large. While 5% of men and 8% of women in the group of upper non-manual employees had new sickness absence spells in 2013, the proportions were 10% and 14% among male and female manual workers, respectively. The proportion was also relatively high (12%) among female lower non-manual employees. In the groups of entrepreneurs and the unemployed, cumulative incidence of sickness absence was at a similar, somewhat lower than average, level. The occupational class of other non-wage-earners showed low cumulative incidence of sickness absence. However, differences in the average number of sickness absence days per one person with incident spells showed quite different associations with occupational class than the incidence of sickness absence did (Table 1). Generally, the number of absence days increased when going from upper non-manual employees towards lower non-manual employees and manual workers. Noteworthy, the number of sickness absence days was substantially higher than average among the entrepreneurs, and especially among the unemployed and in the group of other non-wage-earners. The average number of sickness absence days was 154 days among the unemployed men who had started a new sickness absence spell in 2013, and 137 among the unemployed women, respectively.

Table 2 shows the proportions of those having sickness absence and the number of sickness absence days separately in the two largest diagnostic groups, i.e., musculoskeletal diseases and mental disorders. 2% of men and 3% of women had incident sickness absence spells due to musculoskeletal disorders, and 1% and 2% due to mental disorders, respectively. While in musculoskeletal disorders the average number of sickness absence days was similar as in all-cause sickness absence (Table 1), in mental disorders the number of absence days was substantially larger (118 days among men, 89 among women). Moreover, the general differences between socio-demographic groups were similar in sickness absence due to musculoskeletal diseases as in all-cause sickness absence. Mental disorders slightly diverged from the general pattern: for example, the age group differences were rather small, and lower non-manual employees showed highest incidence of sickness absence. However, concerning the length of sickness absences, the differences between occupational classes were consistent across the examined diagnostic groups, and the spells were longest among the entrepreneurs, the unemployed and other non-wage-earners both in musculoskeletal and in mental disorders (Table 2).

### 3.3. ZINB Modeling Results

Table 3 shows the results of the zero-inflated negative binomial (ZINB) regression models concerning the occupational classes in terms of (a) cumulative incidence, i.e., having any sickness absence (the logit part of the table) and (b) length, i.e., the number of sickness absence days among those who had sickness absence spells (the negative binomial part of the table), by diagnostic group. All socio-demographic covariates were adjusted for in each model. The estimates concerning the covariates are available from the authors by request.

Concerning all-cause sickness absence (all diseases), the employee groups showed a consistent socioeconomic gradient: compared to upper non-manual employees, lower non-manual employees had almost 50% higher odds of having any sickness absence (OR: 1.47 among men, 1.48 among women). The odds of manual workers were even higher, especially among men (OR: 2.13 among men, 1.70 among women). Thus, manual workers showed highest probability of sickness absence also adjusted for covariates. Concerning the non-employee groups, among men, also the entrepreneurs and the unemployed showed about 26–27% higher odds of sickness absence compared to upper non-manual employees. Among women, in contrast, the group of other non-wage-earners had 21% lower odds of sickness absence compared to the upper non-manual employees but the incidence among the entrepreneurs and the unemployed did not differ from that of upper non-manual employees.

In all-cause sickness absence, the negative binomial parts of the ZINB models showed associations partly of different magnitude and partly in the opposite direction. The expected number of sickness absence days were not statistically significantly different when comparing lower non-manuals to upper non-manuals. Among manual workers, the expected number of days was only rather moderately, although statistically significantly, higher than among upper non-manuals (IRR: 1.18 among men, 1.11 among women). However, the entrepreneurs and especially the unemployed showed larger differences compared to upper non-manuals in the expected number of days than in the probability of sickness absence. Among unemployed men, the expected number of sickness absence days was 82% higher compared to upper non-manual employees, and 71% higher among unemployed women, respectively. The group of other non-wage-earners also showed high expected numbers of sickness absence days even though the adjusted odds of having any sickness absence were relatively low in this group.

The association of occupational class with the incidence of sickness absence due to musculoskeletal diseases was stronger than the association with all-cause sickness absence. Both among men and women, the differences in odds in almost all other occupational classes compared to upper non-manual employees were larger than in all-cause sickness absence. Manual workers had 3.46-fold (men) and 2.96-fold (women) odds of sickness absence due to musculoskeletal diseases compared to upper non-manuals. In this disease group, also female entrepreneurs and female unemployed had statistically significantly higher odds of sickness absence than upper non-manuals. Additionally the IRR:s, denoting the differences in expected number of sickness absence days compared to upper non-manuals, were generally higher in musculoskeletal diseases than in all-cause sickness absence among men. Among women, the IRRs were of similar magnitude as in all-cause sickness absence.

Incidence of sickness absence due to mental disorders showed clearly different results than all-cause sickness absence or sickness absence due to musculoskeletal diseases. While lower non-manual employees had 18–19% increased odds of sickness absence compared to upper non-manuals, the odds were not higher among manual workers in this disease group. Entrepreneurs, on the other hand, had clearly lower odds of sickness absence compared to upper non-manuals (OR 0.65 among men, 0.61 among women). Compared to upper non-manuals, unemployed women had slightly higher odds of sickness absence due to mental disorders, but not unemployed men. Finally, concerning the expected number of sickness absence days due to mental disorders, the results were more similar to the results concerning all-cause sickness absence and sickness absence due to musculoskeletal diseases. Differences in length were small between the three employee groups, but the expected number of days was high in the remaining three groups of entrepreneurs, the unemployed and other non-wage-earners. The unemployed had the highest IRRs compared to the reference group (IRR 1.63 among men, 1.74 among women).

Our analyses focused on the incidence and length of new sickness absence spells that started in year 2013 among persons who had not had previous sickness absence compensated by sickness allowance during a one-year retrospective time frame. We conducted the analyses also without this restriction, thus including also persons with prior sickness absence. These supplementary analyses yielded very similar results as those presented above. In these analyses, the proportions of persons having sickness absence were somewhat higher than when focusing the analyses on persons with no prior sickness absence, but the results concerning the associations of occupational class with incidence and length of sickness absence were nearly identical.

## 4. Discussion

### 4.1. Main Findings of the Study

Utilizing large-scale national register-based data on 1.6 million working-aged Finns, this study examined both cumulative incidence and length of sickness absences that started in 2013, looking at the full spectrum of occupational classes. The study included also the entrepreneurs and the unemployed as well as other non-wage-earners in the analyses alongside the hierarchical occupational classes of upper and lower non-manual employees and manual workers.

The main findings of the study can be summarized as follows: (1) Compared to upper non-manual employees, lower non-manual employees and especially manual workers had higher incidence of sickness absence. The incidence was somewhat higher also among male but not female entrepreneurs and unemployed. (2) Among those with any sickness absence spells, especially not being in a wage-earner position was associated with longer sickness absence spells. The unemployed and the entrepreneurs as well as others outside employment had relatively long spells. In addition, manual workers had longer sickness absence spells than upper non-manual employees. (3) The magnitude of the relative differences between occupational classes varied between different diagnoses and between the two measures of sickness absence. Musculoskeletal diseases showed largely similar results as all-cause sickness absence, but the differences between occupational classes were even more pronounced. Relative differences between the wage earner groups of upper non-manual employees, lower non-manual employees and manual workers were smaller in mental disorders than in musculoskeletal diseases or all-cause sickness absence, and lower non-manual employees showed higher incidence. In sickness absence due to mental disorders, entrepreneurs showed smallest incidence, but their spells were long, as were the spells of the unemployed and of other non-wage-earners.

### 4.2. Interpretation of the Results and Comparison to Previous Studies

Our findings largely corroborate earlier findings on the differences in sickness absence between the occupational classes in the employed population, i.e., that upper non-manual employees have lowest incidence and generally lowest number of absence days, and that lower occupational class in the wage earner population is associated with higher incidence and longer duration of absence [13,14,15,16,17,18,19,20,21,22,23]. Differences between occupational classes have been attributed especially to differences in physical working conditions and in health behavior [13,16,17,43] but also partly to psychosocial working conditions, although results of the latter are mixed [13,16,43]. Employees in higher occupational classes may also have more opportunities to adapt their work tasks to better meet their current work ability so that sickness absence is not always needed at least when having only mild symptoms.

Our results concerning different diagnostic groups were also in line with previous studies: there is a strong socioeconomic gradient in sickness absence due to musculoskeletal diseases so that compared to upper non-manual employees, manual workers have a higher incidence and length of sickness absence, and the results concerning lower non-manuals are in between these two groups [20,21,23]. In sickness absence due to mental disorders, the differences between the hierarchical occupational classes were smaller and showed a pattern where lower non-manual employees have higher incidence than the other groups—a result obtained also in previous studies [20,21]. This may be related to jobs in the group of lower non-manual employees being often both physically and mentally demanding with often low decision latitude (i.e., nursing and child-mining) [20].

We obtained also novel results on sickness absence among the entrepreneurs, the unemployed, and other non-wage-earners in comparison to the traditional hierarchical occupational groups of upper and lower non-manual employees and manual workers. We are not aware of previous nationally representative studies that would have included the entrepreneurs and the unemployed in the same analyses with the wage-earner groups and that would have compared the full spectrum of occupational classes in the working-age population. This is largely because most previous studies have utilized data on employed cohorts only.

Concerning entrepreneurs—a group including both self-employed and owners of companies with salaried employees—our results showed that male entrepreneurs had a higher incidence of sickness absence than upper non-manual employees, but the incidence was lower than among lower non-manual employees or manual workers. Among women, entrepreneurs had a similar level of low incidence as upper non-manual employees. The result was nearly similar in musculoskeletal disorders, but in sickness absence due to mental disorders, both male and female entrepreneurs had a clearly lower risk of sickness absence than any of the other studied groups—lower than even upper non-manual employees. Our results are in contrast to those obtained by a Danish study [24] that found that the self-employed had a higher risk of transitioning from work to sickness absence than the wage-earners in general. However, our results are in line with previous knowledge that entrepreneurs are on average in a better mental and somatic health than wage-earners, probably due to selection of healthier individuals into this group but also due to positive health consequences of their active jobs that may be related to higher job control and job demands [44,45].

On the other hand, the sickness absence spells among the entrepreneurs were long compared to the employee population. Thus, health of the entrepreneurs seems to be polarized: on average, they have a rather low incidence of sickness absence but among those who have sickness absence, the diseases tend to be long-term. This seems to be the case especially concerning mental disorders both among male and female entrepreneurs: while the entrepreneurs have a lower incidence than the employee groups, their sickness absence spells are clearly longer than in any of the employee groups. Because of the responsibilities towards their companies and running their businesses, and due to fear of losses of productivity of the company [46], working while sick may be more common among entrepreneurs than among others. Thus, when sickness absence is finally taken, it may take a long time before the ability to work is restored. In addition, sickness absence compensated by a collectively funded sickness benefit may be a valid alternative for guaranteeing income if the company struggles and there is a risk of unemployment, especially concerning the self-employed persons [46]. Furthermore, in the Finnish context, it is possible that since many entrepreneurs are not as often covered by occupational health care as are the employees [47], they have weaker contacts with the health care system and thus may not benefit from effective monitoring of their work ability. Thus, they may receive less support for regaining their work ability and for returning to work. Concerning the employee population, one important role of the occupational health care is to monitor development of sickness absence and to help design individual return-to-work strategies.

The unemployed, on the other hand, are known to be in poorer health than the employed population [25]. Furthermore, those with unemployment background have a greater risk of sickness absence and disability retirement [26,48]. In our study, however, the unemployed did not stand out as a group of especially high cumulative incidence of sickness absence, although unemployed men had a higher incidence than upper non-manual employees did. In all-cause sickness absence and in musculoskeletal disorders, the unemployed showed quite similar cumulative incidence as the entrepreneurs. In mental disorders, cumulative incidence was close to that of upper non-manual employees. One explanation for the rather low cumulative incidence of sickness absence among the unemployed in this study is that also those unemployed who have weakened work ability already have an income through the unemployment benefit. Thus, they may not apply for sickness allowances and thus are not listed as sickness absent, even though sickness allowance can be paid in the Finnish system also to unemployed persons who are not able to seek or accept employment due to health problems. In consequence, their health problems and disability may often go unnoticed, and thus, untreated.

However, when analyzing the length of sickness absence spells, the results concerning the unemployed were quite similar as concerning the entrepreneurs. The expected number of sickness absence days was higher among the unemployed than in any other group in all-cause sickness absence and in sickness absence due to musculoskeletal diseases and in mental disorders, both among men and women. The reasons may be partly similar as among entrepreneurs. The unemployed do not have access to occupational health care and thus monitoring of their work ability may be inadequate. On the other hand, there are indications that since sickness insurance benefits tend to be higher than unemployment benefits, the unemployed may have an incentive to stay longer on sickness absence [49]. Furthermore, staying on sickness benefits may be a preferred option as the sick-listed unemployed do not have to participate in employment-related activation measures. It is also possible that in a same vein as among the entrepreneurs, those unemployed who become sick-listed have serious health problems, which could explain their relatively long absence spells.

We also included in the analyses the remaining group of other non-wage-earners who could not be classified into the above-mentioned groups. This group is very heterogeneous, including, for example, students, those taking care of the household, and other persons for whom a clear socioeconomic status could not be defined. Thus, also the factors determining their sickness absence are varied. In many aspects, however, they are close to the other non-employee groups of unemployed and entrepreneurs, especially in terms of prolongation of sickness absence spells. Moreover, in this group of other non-wage-earners, the explanations for long spells may be related, for example, to lack of support for work ability.

Our analyses focused on new sickness allowance episodes among persons who had not had previous episodes during a one-year retrospective time frame. We conducted the analyses also without this restriction and found that the results were very similar. Thus, our conclusions are not dependent on this definition of the study cohort.

### 4.3. Strengths and Weaknesses of the Study

Our study had various strengths. A key strength was that we were able to utilize a large-scale, fully representative register-based data set that comprised 70% of working-age Finns, including 1.6 million persons. National registers are deemed to be highly reliable and objective, data quality is high, and there is very little missing information. Also, there is no self-report bias and no loss to follow-up in register data. We were able to measure sickness absence spells very precisely, including their beginning and end dates and diagnoses certified by a physician. Our study aimed to give a comprehensive picture of the socioeconomic differences in sickness absence, and thus both incidence and length of absence were analyzed simultaneously. Another strength was that in contrast to most previous studies, we examined the total working-age population susceptible to sickness absence and sickness allowance, i.e., also the classes of self-employed and unemployed and others without employment. The results may be generalized with some caution also to other countries with a roughly similar social security system, such as Sweden.

The study also had some limitations. The definition of sickness absence in this study is based on receipt of sickness allowance and, hence, refers to rather long-term absence episodes lasting more than 10 working days. Consequently, sickness absences measured in this study tended to be rather long, since shortest spells lasting up to 10 working days could not be observed in the data. Unfortunately, there is no national register data in Finland on shorter sickness absence spells, i.e., spells shorter than 10 working days that do not entitle to sickness allowance. Another limitation of the study is the observational cross-sectional design; thus, no causal effects can be established. Health-related selection into the occupational classes may explain part of the results. Additionally, due to limitations in the availability of different measures in register data, we had no information on several factors that could partly explain the association between socioeconomic class and sickness absence, such as differences in health and functional capacity, different physical and psychosocial working conditions, living conditions, health behavior and use of health services. Previous studies have shown that some of these factors may contribute to the occupational class differences in sickness absence [16,17,43] although their role in explaining the results concerning the situation of the entrepreneurs, the unemployed, and other non-wage-earners remains unclear.

### 4.4. Policy Implications and Future Research

Reducing sickness absence entails two steps: first, preventing the occurrence of absence episodes, and second, preventing the prolongation of episodes that have already started. Especially long sickness absence is a strong predictor of permanently leaving the work force through disability retirement [8,9,10,11]. Great savings on the direct and indirect costs of work disability could be achieved through maintaining work ability in the working-age population and preventing long sickness absences.

Reducing sickness absence may necessitate actions related to, for example, promoting good health and beneficial health behavior, improving physical and psychosocial working conditions and facilitating access to health care. In order to reduce occurrence of sickness absence due to musculoskeletal diseases, preventive measures should be targeted particularly at manual workers’ working conditions that often include heavy physical work tasks. On the other hand, psychosocial working conditions—particularly of lower non-manual employees—should be improved in order to decrease sickness absence due to mental disorders.

In terms of prolongation of absences, the unemployed and the entrepreneurs should be under special focus. Maintaining and improving work ability of the unemployed persons is crucial since they constitute an important labor reserve in the era of a worsening economic dependency ratio. On the other hand, supportive measures should be targeted also at the entrepreneurs. They are often seen to constitute the economic basis of the economy in terms of creating jobs and welfare, and thus also need work ability related support. While employers in Finland are obliged by law to organize occupational health care for employees, the proportion of self-employed that organize occupational health care for themselves is low [47]. Increased efforts from the social and health care system are needed in order to tackle the emerging work ability problems of both entrepreneurs, on the one hand, and of the unemployed, on the other. In particular, regular screening of work ability should be offered by the public health services to the unemployed and entrepreneurs. Earlier diagnosis and timely treatment may help to reduce the length of absence episodes among the wage-earner as well as the non-wage-earner groups.

Further research should focus on better understanding the mechanisms that contribute to social inequalities in sickness absence and focus more specifically on different diagnoses. Some previous research exists on factors that could explain differences between the occupational classes in employed populations [13,16,17,43], but studies are needed on the factors contributing to the long duration of sickness absence spells among the entrepreneurs and the unemployed. Additionally, intervention studies are needed on actions that aim to shorten sickness absence spells, especially concerning the non-wage-earner groups who are at a risk of prolongation of absences due to lack of effective monitoring. For example, studies could investigate whether a more vigorous functioning of employment services in referring the unemployed to health services for health check-ups would help spot and tackle their work ability issues early and reduce the length of sickness absences.

Furthermore, more research on sickness absence among both the wage-earner and non-wage-earner groups is needed from other countries, such as from the Scandinavian welfare states having roughly similar social security systems and data sources available for research. Later research needs to ascertain whether the results observed in this study hold also in other countries.

## 5. Conclusions

There were large differences between upper and lower non-manual employees, manual workers, entrepreneurs, the unemployed and other non-wage-earners in both the cumulative incidence and length of sickness absence. The magnitude of differences varied according to diagnostic group and measure of sickness absence. In order to curb costs and harms related to sickness absence, actions are needed to reduce occurrence of sickness absence, on the one hand, and to prevent prolongation of absence spells, on the other. While manual workers were the group with highest cumulative incidence of sickness absence in general, the unemployed and entrepreneurs, as well as other non-wage-earners, stood out as groups with a high risk or prolongation of sickness absence spells. Further research is needed on the explanations for the observed differences and of potential intervention strategies to reduce both occurrence and prolongation of absences. It is important to understand different patterns of sickness absence in different socioeconomic groups to be able to target effective preventive measures accordingly. Diverse measures are needed in order to reduce the overall burden of sickness absence in the working-age population and to narrow differences between the occupational classes. Different socioeconomic groups may benefit from different policies and preventive measures, notably since those in employment may utilize occupational health care. Special efforts should be targeted to maintaining work ability also in non-wage-earner groups, such as the unemployed and entrepreneurs, who are often not subject to specific work ability monitoring and support by occupational health care to prevent unnecessary prolongation of sickness absence spells. Preventing the accumulation of sickness absence days is crucial also in reducing disability retirement and permanent early withdrawal from the labor market. Measures are needed to combat both the incidence and prolongation of sick leave in order to save costs and prevent the loss of potential labor input.

## Figures and Tables

**Table 1:** Distributions of the study population in the end of 2012, proportion (%) of persons having all-cause sickness absence (SA) in 2013 and mean number of sickness absence days among persons with sickness absence spells.

Covariate	Distribution (%)		Proportion (%) Having SA		Mean Number of SA Days ^1^	
	Men	Women	Men ^2^	Women ^2^	Men ^2^	Women ^2^
	N = 822,766	N = 792,586	N = 59,086	N = 81,830	N = 59,086	N = 81,830
**Age**						
25–34 years	28.2	27.2	5.3	8.0	70	61
35–44 years	26.1	25.4	6.5	9.5	74	64
45–54 years	27.5	27.8	8.4	11.9	86	72
55–62 years	18.3	19.6	9.3	12.4	105	85
**Marital status**						
Married	49.6	53.8	7.5	10.3	79	69
Never married	39.3	31.0	6.1	8.9	86	69
Divorced or widowed	11.1	15.2	9.2	13.2	98	79
**Region**						
Capital and Uusimaa region	31.5	32.7	5.9	8.6	80	66
Southern Finland	21.3	21.4	7.7	10.6	83	73
Western Finland	24.7	24.0	7.5	11.1	84	71
Northern and Eastern Finland	22.5	21.9	8.1	11.8	90	75
**Income quintile**						
Highest	27.3	12.4	6.4	8.3	62	57
Second highest	22.4	17.5	8.7	11.3	71	58
Middle	17.2	23.0	8.9	13.0	78	61
Second lowest	14.8	25.4	7.0	11.8	102	74
Lowest	18.3	21.7	5.1	6.1	147	118
**Occupational class**						
Upper non-manual	19.2	19.7	4.9	7.9	59	57
Lower non-manual	18.6	42.9	7.0	12.4	63	62
Manual worker	30.9	14.3	10.2	13.5	77	73
Entrepreneur	12.8	6.9	6.4	7.9	97	85
Unemployed	11.6	8.8	6.0	7.2	154	137
Other	6.9	7.3	4.0	4.9	144	122
All	100	100	7.2	10.3	84	71

^1^ Average total length of absence among those with incident sickness absence compensated by sickness allowance. ^2^ N refers to the number of persons who had any sickness absence spells.

**Table 2:** Proportion of persons having sickness absence (SA) in 2013 and mean number of sickness absence days among person with sickness absence spells due to musculoskeletal diseases and mental disorders by covariates.

Covariate	Musculoskeletal Diseases				Mental Disorders			
	Proportion (%) Having SA		Mean Number of SA Days ^1^		Proportion (%) Having SA		Mean Number of SA Days ^1^	
	Men ^2^	Women ^2^	Men ^2^	Women ^2^	Men ^3^	Women ^3^	Men ^3^	Women ^3^
	N = 19,248	N = 26,563	N = 19,248	N = 26,563	N = 7372	N = 14,944	N = 7372	N = 14,944
**Age**								
25–34 years	1.3	1.9	65	57	1.0	1.8	109	91
35–44 years	2.1	2.8	70	62	0.9	2.1	112	82
45–54 years	3.0	4.2	85	73	0.9	2.0	125	90
55–62 years	3.3	4.9	110	87	0.7	1.5	142	95
**Marital status**								
Married	2.6	3.5	83	73	0.8	1.7	103	82
Never married	1.8	2.5	83	65	1.0	1.9	130	93
Divorced or widowed	3.1	4.7	96	78	1.2	2.6	131	98
**Region**								
Capital and Uusimaa	1.8	2.6	79	67	0.8	1.7	112	84
Southern Finland	2.6	3.6	83	74	0.9	1.8	117	89
Western Finland	2.5	3.7	85	71	1.0	2.1	120	89
Northern and Eastern Finland	2.7	4.0	92	77	1.0	2.0	125	94
**Income quintile**								
Highest	1.9	2.1	61	59	0.7	1.4	81	67
Second highest	3.1	3.5	73	58	0.8	2.0	81	64
Middle	3.3	4.5	80	63	0.9	2.2	87	64
Second lowest	2.3	4.3	111	76	1.0	2.0	122	86
Lowest	1.2	1.7	148	122	1.2	1.6	198	163
**Occupational class**								
Upper non-manual	1.2	1.8	55	57	0.8	1.7	83	68
Lower non-manual	2.0	4.0	64	64	1.0	2.2	80	67
Manual worker	4.1	5.9	78	72	0.9	1.8	90	83
Entrepreneur	1.9	2.3	106	91	0.6	1.1	140	132
Unemployed	1.6	2.1	156	143	1.2	1.9	204	178
Other	0.9	1.2	146	115	1.2	1.7	195	169
All	2.3	3.4	85	72	0.9	1.9	118	89

^1^ Average total length of absence due to the diagnostic group among those with incident sickness absence compensated by sickness allowance. ^2^ N refers to the number of persons who had sickness absence spells due to musculoskeletal diseases. ^3^ N refers to the number of persons who had sickness absence spells due to mental disorders.

**Table 3:** Zero-inflated negative binomial (ZINB) regression analyses: the association of occupational class with the cumulative incidence of sickness absence (SA) and length of sickness absence, adjusted for covariates.

	Men				Women			
	Logit Part: Incidence of SA		Negative Binomial Part: Length of SA		Logit Part: Incidence of SA		Negative Binomial Part: Length of SA	
	OR ^1^	95% CI	IRR ^2^	95% CI	OR ^1^	95% CI	IRR ^2^	95% CI
**All diseases**								
Upper non-manual	1.00		1.00		1.00		1.00	
Lower non-manual	**1.47**	**1.43–1.52**	1.01	0.98–1.05	**1.48**	**1.44–1.51**	1.02	0.99–1.05
Manual worker	**2.13**	**2.07–2.19**	**1.18**	**1.14–1.22**	**1.70**	**1.65–1.74**	**1.11**	**1.07–1.14**
Entrepreneur	**1.26**	**1.21–1.30**	**1.33**	**1.27–1.38**	1.02	0.98–1.06	**1.23**	**1.17–1.28**
Unemployed	**1.27**	**1.22–1.32**	**1.82**	**1.74–1.90**	0.98	0.95–1.02	**1.71**	**1.64–1.78**
Other	1.04	0.99–1.10	**1.78**	**1.68–1.88**	**0.79**	**0.76–0.83**	**1.63**	**1.55–1.72**
**Musculoskeletal diseases**								
Upper non-manual	1.00		1.00		1.00		1.00	
Lower non-manual	**1.78**	**1.67–1.89**	**1.08**	**1.08–1.16**	**1.96**	**1.88–2.05**	1.06	1.00–1.11
Manual worker	**3.46**	**3.27–3.66**	**1.27**	**1.19–1.35**	**2.96**	**2.82–3.11**	**1.09**	**1.02–1.15**
Entrepreneur	**1.49**	**1.40–1.60**	**1.50**	**1.39–1.62**	**1.27**	**1.18–1.36**	**1.31**	**1.21–1.42**
Unemployed	**1.56**	**1.44–1.68**	**1.94**	**1.79–2.11**	**1.21**	**1.13–1.30**	**1.74**	**1.61–1.88**
Other	**1.22**	**1.09–1.35**	**1.93**	**1.73–2.15**	0.94	0.86–1.03	**1.54**	**1.39–1.71**
**Mental disorders**								
Upper non-manual	1.00		1.00		1.00		1.00	
Lower non-manual	**1.18**	**1.09–1.27**	0.91	0.82–1.00	**1.19**	**1.13–1.25**	0.94	0.89–1.00
Manual worker	0.95	0.88–1.03	1.01	0.92–1.11	0.94	0.88–1.00	1.03	0.95–1.11
Entrepreneur	**0.65**	**0.59–0.72**	**1.38**	**1.24–1.53**	**0.61**	**0.56–0.67**	**1.56**	**1.41–1.72**
Unemployed	1.10	1.00–1.20	**1.63**	**1.48–1.81**	**1.09**	**1.01–1.17**	**1.74**	**1.60–1.89**
Other	1.04	0.94–1.16	**1.59**	**1.43–1.78**	0.99	0.91–1.07	**1.67**	**1.53–1.82**

^1^ Logistic regression part from the ZINB model: analysis of cumulative incidence of sickness absence, calculated as inverted odds ratios to denote the odds of being in the non-zero group. Adjusted for age, marital status, region of residence and income. Estimates in bold are statistically significant using the significance level of *p* < 0.05. ^2^ Negative binomial regression part from the ZINB model: analysis of the length of SA, i.e., number of absence days among persons with incident sickness absence. Adjusted for age, marital status, region of residence and income. Estimates in bold are statistically significant using the significance level of *p* < 0.05.

## Data Availability

Due to legal restrictions and data protection regulations of the administrative sources providing individual-level register data, the authors do not have the permission to make sensitive personal data available. Interested parties may apply for permissions to access the data from the centralized data permit authority Findata (https://www.findata.fi/en/) and from Statistics Finland (https://www.stat.fi/tup/mikroaineistot/hakumenettely_en.html).

## References

[1] Henderson M., Glozier N., Holland Elliott K. (2005). Long Term Sickness Absence. BMJ.

[2] OECD (2010). Sickness, Disability and Work: Breaking the Barriers. A Synthesis of Findings across OECD Countries.

[3] Sutela H., Pärnänen A., Keyriläinen M. (2019). Digiajan Työelämä—Työolotutkimuksen Tuloksia 1977–2018 (Working Life of the Digital Era—Results of the Working Condition Surveys 1977–2018, in Finnish Official Statistics Finland).

[4] Elinkeinoelämän Keskusliitto (2016). Työaikakatsaus. Työajat ja Poissaolot EK:n Jäsenyrityksissä Vuonna 2014 (Overview of the Working Time. Working Hours and Absences in EK Member Companies in 2014, in Finnish).

[5] Blomgren J. (2016). Pitkät Sairauspoissaolot Työikäisillä Naisilla ja Miehillä. Sairauspäivärahan Saajat 1996–2015 (Long Sickness Absences among Working-Aged Women and Men. Recipients of Sickness Allowance 1996–2015, in Finnish). Yhteiskuntapolitiikka.

[6] Marmot M., Freeney A., Shipley M., North F., Syme S.L. (1995). Sickness Absence as a Measure of Health Status and Functioning: From the UK Whitehall II Study. J. Epidemiol. Community Health.

[7] Vahtera J., Pentti J., Kivimäki M. (2004). Sickness Absence as a Predictor of Mortality among Male and Female Employees. J. Epidemiol. Community Health.

[8] Kivimäki M., Forma P., Wikström J., Halmeenmäki T., Pentti J., Elovainio M., Vahtera J. (2004). Sickness Absence as a Risk Marker of Future Disability Pension: The 10-Town Study. J. Epidemiol. Community Health.

[9] Alexanderson K., Kivimäki M., Ferrie J.E., Westerlund H., Vahtera J., Singh-Manoux A., Melchior M., Zins M., Goldberg M., Head J. (2012). Diagnosis-Specific Sick Leave as a Long-Term Predictor of Disability Pension: A 13-Year Follow-Up of the GAZEL Cohort Study. J. Epidemiol. Community Health.

[10] Salonen L., Blomgren J., Laaksonen M., Niemelä M. (2018). Sickness Absence as a Predictor of Disability Retirement in Different Occupational Classes: A Register-Based Study of a Working-Age Cohort in Finland in 2007–2014. BMJ Open.

[11] Salonen L., Blomgren J., Laaksonen M. (2020). From Long-Term Sickness Absence to Disability Retirement: Diagnostic and Occupational Class Differences within the Working-Age Finnish Population. BMC Public Health.

[12] Allebeck P., Mastekaasa A., Swedish Council on Technology Assessment in Health Care (SBU) (2004). Chapter 5. Risk Factors for Sick Leave—General Studies. Scand. J. Public Health.

[13] Christensen K.B., Labriola M., Lund T., Kivimäki M. (2008). Explaining the Social Gradient in Long-Term Sickness Absence: A Prospective Study of Danish Employees. J. Epidemiol. Community Health.

[14] Hansen H., Ingebrigtsen T. (2008). Social Class and Sickness Absence in Norway. Acta Sociol..

[15] Kristensen T.R., Jensen S.M., Kreiner S., Mikkelsen S. (2010). Socioeconomic Status and Duration and Pattern of Sickness Absence. A 1-Year Follow-Up Study of 2331 Hospital Employees. BMC Public Health.

[16] Laaksonen M., Piha K., Rahkonen O., Martikainen P., Lahelma E. (2010). Explaining Occupational Class Differences in Sickness Absence: Results from Middle-Aged Municipal Employees. J. Epidemiol. Community Health.

[17] Löve J., Hensing G., Holmgren K., Torén K. (2013). Explaining the Social Gradient in Sickness Absence: A Study of a General Working Population in Sweden. BMC Public Health.

[18] Sumanen H., Lahelma E., Pietiläinen O., Rahkonen O. (2017). The Magnitude of Occupational Class Differences in Sickness Absence: 15-Year Trends among Young and Middle-Aged Municipal Employees. Int. J. Environ. Res. Public Health.

[19] Pekkala J., Blomgren J., Pietiläinen O., Lahelma E., Rahkonen O. (2017). Occupational Class Differences in Long Sickness Absence: A Register-Based Study of 2.1 Million Finnish Women and Men in 1996–2013. BMJ Open.

[20] Pekkala J., Blomgren J., Pietiläinen O., Lahelma E., Rahkonen O. (2017). Occupational Class Differences in Diagnostic-Specific Sickness Absence: A Register-Based Study in the Finnish Population, 2005–2014. BMC Public Health.

[21] Leinonen T., Viikari-Juntura E., Husgafvel-Pursiainen K., Solovieva S. (2018). Cause-Specific Sickness Absence Trends by Occupational Class and Industrial Sector in the Context of Recent Labour Market Changes: A Finnish Panel Data Study. BMJ Open.

[22] Airaksinen J., Jokela M., Virtanen M., Oksanen T., Koskenvuo M., Pentti J., Vahtera J., Kivimäki M. (2018). Prediction of Long-Term Absence Due to Sickness in Employees: Development and Validation of a Multifactorial Risk Score in Two Cohort Studies. Scand. J. Work Environ. Health.

[23] Pekkala J., Rahkonen O., Pietiläinen O., Lahelma E., Blomgren J. (2018). Sickness Absence Due to Different Musculoskeletal Diagnoses by Occupational Class: A Register-Based Study among 1.2 Million Finnish Employees. Occup. Environ. Med..

[24] Pedersen J., Bjorner J.B., Burr H., Christensen K.B. (2012). Transitions between Sickness Absence, Work, Unemployment, and Disability in Denmark 2004–2008. Scand. J. Work Environ. Health.

[25] McKee-Ryan F., Song Z., Wanberg C.R., Kinicki A.J. (2005). Psychological and Physical Well-Being during Unemployment: A Meta-analytic Study. J. Appl. Psychol..

[26] Helgesson M., Johansson B., Nordqvist T., Lundberg I., Vingard E. (2013). Unemployment at a Young Age and Later Sickness Absence, Disability Pension and Death in Native Swedes and Immigrants. Eur. J. Public Health.

[27] Finnish National Board on Research Integrity TENK (2019). The Ethical Principles of Research with Human Participants and Ethical Review in the Human Sciences in Finland.

[28] Toivonen L. (2012). Statutory and Occupational Sickness Benefits in Finland in 2011.

[29] World Health Organization (2016). World Health Organization ICD-10. International Statistical Classification of Diseases and Related Health Problems, 10th Revision.

[30] Statistics Finland Classification of Socio-Economic Groups 1989. https://www.stat.fi/en/luokitukset/sosioekon_asema/sosioekon_asema_1_19890101.

[31] Hensing G. (2009). The Measurements of Sickness Absence—A Theoretical Perspective. Nor. Epidemiol..

[32] Lehnert T., Stuhldreher N., Streltchenia P., Riedel-Heller S.G., König H. (2014). Sick Leave Days and Costs Associated with Overweight and Obesity in Germany. J. Occup. Environ. Med..

[33] Taimela S., Läärä E., Malmivaara A., Tiekso J., Sintonen H., Justén S., Aro T. (2007). Self-Reported Health Problems and Sickness Absence in Different Age Groups Predominantly Engaged in Physical Work. Occup. Environ. Med..

[34] Vuong Q.H. (1989). Likelihood Ratio Tests for Model Selection and Non-Nested Hypotheses. Econometrica.

[35] Long J.S., Freese J. (2014). Regression Models for Categorical Dependent Variables Using Stata.

[36] Afifi A.A., Kotlerman J., Ettner S.L., Cowan M. (2007). Methods for Improving Regression Analysis for Skewed Continuous or Counted Responses. Annu. Rev. Public Health.

[37] Cameron A.C., Trivedi P.K. (2010). Microeconometrics Using Stata. Revised Edition.

[38] Greene W. (2012). Econometric Analysis.

[39] Asfaw A.G., Chang C.C., Ray T.K. (2014). Workplace Mistreatment and Sickness Absenteeism from Work: Results from the 2010 National Health Interview Survey. Am. J. Ind. Med..

[40] Catalina-Romero C., Sainz J.C., Pastrana-Jiménez J.I., García-Diéguez N., Irízar-Muñoz I., Aleixandre-Chiva J.L., Gonzalez-Quintela A., Calvo-Bonacho E. (2015). The Impact of Poor Psychosocial Work Environment on Non-Work-Related Sickness Absence. Soc. Sci. Med..

[41] Hilbe J.M. (2011). Negative Binomial Regression.

[42] (2013). StataCorp Stata Statistical Software: Release 13.

[43] Melchior M., Krieger N., Kawachi I., Berkman L.F., Niedhammer I., Goldberg M. (2005). Work Factors and Occupational Class Disparities in Sickness Absence: Findings from the GAZEL Cohort Study. Am. J. Public Health.

[44] Stephan U., Roesler U. (2010). Health of Entrepreneurs Versus Employees in a National Representative Sample. J. Occup. Organ. Psychol..

[45] Herber G., Schipper M., Koopmanschap M., Proper K., van der Lucht F., Boshuizen H., Polder J., Uiters E. (2020). Health Expenditure of Employees Versus Self-Employed Individuals; A 5 Year Study. Health Econ..

[46] Spierdijk L., van Lomwel G., Peppelman W. (2009). The Determinants of Sick Leave Durations of Dutch Self-Employed. J. Health Econ..

[47] Lappalainen K., Aminoff M., Hakulinen H., Hirvonen M., Räsänen K., Sauni R., Stengård J. (2016). Työterveyshuolto Suomessa 2015 ja Kehitystrendi 2000–2015 (Occupational Health Care in Finland 2015 and Trend in 2000–2015).

[48] Laaksonen M., Blomgren J. (2020). The Level and Development of Unemployment before Disability Retirement: A Retrospective Study of Finnish Disability Retirees and Their Controls. Int. J. Environ. Res. Public Health.

[49] Engström L., Eriksen T. (2002). Can Differences in Benefit Levels Explain Duration and Outcome of Sickness Absence?. Disabil. Rehabil..

